# Microstructure, Texture and Mechanical Properties of Mg-6Sn Alloy Processed by Differential Speed Rolling

**DOI:** 10.3390/ma14010083

**Published:** 2020-12-26

**Authors:** Kamil Majchrowicz, Paweł Jóźwik, Witold Chromiński, Bogusława Adamczyk-Cieślak, Zbigniew Pakieła

**Affiliations:** 1 Faculty of Materials Science and Engineering, Warsaw University of Technology, Woloska 141, 02-507 Warsaw, Poland; witold.chrominski@pw.edu.pl (W.C.); boguslawa.cieslak@pw.edu.pl (B.A.-C.); zbigniew.pakiela@pw.edu.pl (Z.P.); 2 Faculty of Advanced Technologies and Chemistry, Military University of Warsaw, Kaliskiego 2, 00-908 Warsaw, Poland

**Keywords:** Mg-Sn alloys, differential speed rolling, microstructure, texture, EBSD, basal texture splitting, low anisotropy

## Abstract

The effect of shear deformation introduced by differential speed rolling (DSR) on the microstructure, texture and mechanical properties of Mg-6Sn alloy was investigated. Mg-6Sn sheets were obtained by DSR at speed ratio between upper and lower rolls of R = 1, 1.25, 2 and 3 (R = 1 refers to symmetric rolling). The microstructural and textural changes were investigated by electron backscattered diffraction (EBSD) and XRD, while the mechanical performance was evaluated based on tensile tests and calculated Lankford parameters. DSR resulted in the pronounced grain refinement of Mg-6Sn sheets and spreading of basal texture as compared to conventionally rolled one. The average grain size and basal texture intensity gradually decreased with increasing speed ratio. The basal poles splitting to transverse direction (TD) or rolling direction (RD) was observed for all Mg-6Sn sheets. For the as-rolled sheets, YS and UTS increased with increasing speed ratio, but a significant anisotropy of strength and ductility between RD and TD has been observed. After annealing at 300 °C, Mg-6Sn sheets became more homogeneous, and the elongation to failure was increased with higher speed ratios. Moreover, the annealed Mg-6Sn sheets were characterized by a very low normal anisotropy (0.91–1.16), which is normally not achieved for the most common Mg-Al-Zn alloys.

## 1. Introduction

The main reason of the limited usage of wrought magnesium alloys in structural applications is their low ductility and poor formability that produce higher costs and difficulties in their forming processes [1,2]. Mg alloys with the low symmetry hexagonal close-packed structure show limited formability due to unsatisfactory number of independent slip systems. At room temperature dislocation slip in Mg occurs mainly on the densely packed basal (0001) planes along the <11–20> directions. Since basal slip mode provides only two independent slip systems and cannot meet the Von Mises criterion for arbitrary shape change [3], additional slip or twin systems have to be activated [4,5,6].

One of the approaches to increase formability of Mg is the use of alloying elements, which decrease stacking fault energy (SFE) of Mg [5,6,7]. Muzyk et al. [8] has proven by the density functional theory (DFT) that the so-called generalized stacking fault energy (GSFE) and tendency to partial dislocation emission varies with different alloying additives. Sn strongly reduces the GSFE of Mg in the basal {0001}<1120> and prismatic {1–100}<−1–120> slip systems [8]. The reports by Wang et al. [9] and Zhang et al. [10] showed also that Sn atoms significantly lower the GFSE in the pyramidal {10–11}<11–20> and {11–22}<11–23> slip modes as well. Moreover, Zeng et al. [11] has recently calculated the theoretical critical shear strength for basal <a> and pyramidal <c+a> slip systems, which reflects the value of critical resolved shear stress (CRSS). After doping Sn to pure Mg, the critical shear strength in the basal slip raised from 114 to 149 MPa, whereas it was reduced drastically from 1571 to 446 MPa for the pyramidal slip. Thus, it has been observed that Sn may activate those slip systems, which normally are unfavored leading to the improvement of ductility and formability of Mg-Sn alloys.

Theoretical assumptions have been proved by some researchers for a dilute (up to 1 wt.%) [11,12,13,14] as well as higher Sn contents (5–8 wt.%) [15,16,17]. The extruded and hot rolled Mg-0.4Sn-0.7Y alloy sheets investigated by Wang et al. [12,13] exhibited high ductility (around 33 and 32%, respectively) which has been attributed to the significant texture weakening and basal texture splitting. Suh et al. [14] showed also that the small addition of Sn (1 wt.%) instead of Zn to Mg-3Al alloy improves its stretch formability due to uniform deformation and activation of the prismatic <a> slip. The formability of Mg-Sn alloys can be improved by raising Sn content as well. Zeng et al. [11] confirmed that the rollability of solutionized Mg-Sn alloys (with varying Sn addition from 0.5 to 3.5 wt.%) and the ductility of as-extruded Mg-Sn sheets increase with increasing amount of Sn. Higher Sn content (5–8 wt.%) is still sufficient to obtain Mg alloy with a good combination of strength and formability [15,16,17]. Yoon and Park [15] observed that Mg-Sn-based alloys (TAZ541, TAZ711, TAZ811) exhibit superior forgeability at elevated forming temperature as compared to conventional Mg-Al-Zn alloys (AZ61, AZ80). All those results indicate that Mg-Sn-based alloys are a promising group with potentially enhanced ductility and formability.

Conventional symmetric rolling generally gives rise to a strong basal texture {0001}<10–10>. This induces a high normal anisotropy in Mg sheets and leads to their limited formability at further processing steps [2,4]. Since the formability of Mg is strongly affected by the basal texture, it can be improved by reduction in texture intensity by introducing intense shear deformation [5,6,18]. Differential speed rolling (DSR) is an industrial scale modification of sheets rolling process that introduces intense shear deformation throughout the sheet thickness by controlled differentiation of rotation speeds for upper and lower rolls [18,19,20]. It has been shown for AZ31 Mg alloy that this method is very effective to obtain Mg sheets with enhanced ductility due to microstructural and textural modification [21,22]. Compared with the normal symmetrically rolled sheet, the DSR processed sheets exhibit increased uniform elongation and strain hardening exponent, while the anisotropy of strength and plasticity (expressed as a Lankford value) is decreased, especially in the rolling direction [18,21]. The enhanced ductility is achieved by a weakening of a basal texture intensity and inclinations of basal poles up to even 15° leading to easier {10–12} extension twinning and (0001)<11–20> dislocation slip [21]. Moreover, the DSR processed AZ31 alloy sheets exhibit superior formability in comparison with symmetrically rolled ones. The Erichsen values of the DSR processed sheet was improved from 2.6 to 4.0 and 4.1 to 7.6 at room temperature and 150 °C, respectively [18].

Up to now, several processing methods, which induce the intense shear strain in the whole deformed parts, including equal channel angular extrusion (ECAE) [23], cross-roll rolling [24] and DSR [18,21,22], have been conducted on the Mg alloys. Among these processing methods, the DSR shows a high potential to meet industrial needs by the capability of forming large thin parts without the necessity of using complex roll shape and other equipment. It has been proved that DSR is a highly effective process to enhance ductility of Mg-Al-Zn [18,21,22], Mg-Zn-Zr [25] and Mg-Al-Mn [26] alloys, but to the our best knowledge, there is no literature mentions about Mg-Sn alloys processed by DSR. Thus, the aim of the present work was to investigate the effect of the shear deformation introduced by DSR processing on the microstructure, texture and mechanical properties of Mg-6Sn alloy (wt.%). The level of introduced shear stresses during DSR was controlled by adjusting different speed ratio (R) between upper and lower rolls, i.e., R = 1, 1.25, 2 and 3. The symmetrically rolled Mg-6Sn sheets (R = 1) were used as a reference material.

## 2. Materials and Methods

### 2.1. Material Preparation

The material used in this study was a Mg-6Sn alloy with the exact Sn content of 6.02 wt.% measured by atomic absorption spectrometry. The as-received samples were in the form of φ25 mm hot extruded rods. Before rolling, the rods were machined into rectangular slabs with dimensions of 200 × 20 × 10 mm and solution heat treated at 480 °C for 1 h followed by water quenching. Rolling was performed on a sexton type laboratory mill stand equipped with working rolls made of tungsten carbide having an equal diameter of 85 mm and a length of 150 mm. The slabs were preheated at 400 °C for 10 min and then, subjected to 4 passes with a reduction ratio of 15% per pass. No lubrication was applied during rolling. After each pass, the rolled specimens were reheated at 400 °C for 10 min and rotated by 180° around the rolling direction. Such a DSR rolling route of Mg alloys is the most effective one to obtain: an equiaxed, homogeneous structure with a weak basal texture due to the intersecting of shear bands during the consecutive rolling passes [26,27]. The speed ratio R (the ratio between upper and lower rolls) varied from 1 to 1.25, 2 and 3 with the constant velocity of the upper roll maintained at 4 m/min. As mentioned earlier, R = 1 corresponds to conventional symmetric rolling with equal speed of both rolls. According to the literature reports, DSR process with a low speed asymmetry (R < 1.5) should result mainly in the basal texture modification of Mg alloys [18,21,22,28], whereas higher speed asymmetry ratios (R ≥ 2) may cause a significant grain refinement as well [27,28]. Mg-6Sn sheets were investigated in the as-rolled state and after further annealing at 300 °C for 1 h.

### 2.2. Material Examination

The microstructure of as-rolled sheets was characterized in terms of grain size, grain boundaries characteristics and microtexture by electron backscattered diffraction (EBSD) method using Hitachi SU-70 scanning electron microscope (SEM, Hitachi, Tokyo, Japan) equipped with a Schottky emitter. EBSD measurements were performed on the rolling direction/normal direction (RD-ND) plane in the middle of the sheet thickness. The samples for EBSD were prepared by grinding and ion polishing using Hitachi IM4000 Ion Milling System. Maps were taken with an acceleration voltage of 20 kV from the area of at least 100,000 μm^2^ using a step size of 0.4 μm. Each map had a ratio of successfully indexed Kikuchi maps > 65%, thus data were considered reasonable. Data were analyzed with a dedicated HKL Channel 5 software (version 5.0). The grain size was expressed by the equivalent diameter, which equals to the diameter of a circle with the same area as the grain under consideration. The grain boundaries characteristic was determined assuming the misorientation angle of 3 to 15° for low angle grain boundaries (LAGBs, marked as faint grey lines on EBSD maps) and above 15° for high angle grain boundaries (HAGBs, marked as black lines). Misorientation angles lower than 3° were not taken into account due to a substantial “orientation noise” in heavily deformed materials [29].

Metallographic observations of annealed sheets were performed with a Zeiss Axio Observer light microscope (LM, Zeiss, Oberkochen, Germany). The RD-ND cross-sections for microscopic examination were ground up to 4000 grit SiC paper, polished using 3 and 1 μm ethanol-based diamond suspensions and etched with a solution of 2% nital. Based on the LM images, the mean grain size was described by the equivalent diameter measured by the MicroMeter software [30,31].

The texture and phase composition were determined by X-ray diffraction (XRD, Bruker, Billerica, USA) using Bruker D8 Discover diffractometer with Co Kα radiation (λ = 1.79 Å). Diffraction patterns were recorded with a step of 0.02° and counting time of 5 s on the rolling direction/transverse direction (RD-TD) plane in the middle of the sheet thickness. Four incomplete pole figures, i.e., (0002), (10–10), (10–11) and (10–12), were used to calculate the orientation distribution functions (ODFs) and recalculate complete pole figures using LaboTex software. Bunge notation of the Euler angles (ϕ_1_, Φ, ϕ_2_) was introduced to represent the ODFs.

To evaluate mechanical properties and plastic anisotropy of the DSR-processed sheets, uniaxial tensile tests were conducted on a static testing machine Zwick/Roell Z005 equipped with a 1 kN load cell. The miniaturized tensile samples with a gauge length of 10 mm and a cross-section of 1.6 × 1.2 mm (described more in detail in [32,33]) were cut at angles of 0, 45 and 90° to the RD. Three samples through the sheet thickness were obtained for each direction. Tensile tests were performed at initial strain rate of 10^−3^ s^−1^ using a Digital Image Correlation (DIC) method for strain analysis [34]. DIC measurements were conducted on the front and side surfaces of tensile samples, which allowed to accurately calculate the Lankford parameters (r_α_ values where α indicates an angle to the RD). This parameter is defined as a ratio of true strains along the width (ε_w_) to thickness (ε_t_) of the tensile sample at a given elongation between yield and ultimate tensile strength. In this work, the Lankford values were measured at a strain of 5%. Additionally, the normal anisotropy r_m_, planar anisotropy Δr and earing tendency Δr_p_ were calculated using the following formulas [35]:

(1) $$r_{m}=\;\left(r_{0}+\;2r_{45}+{\;r}_{90}\right)/4$$

(2) $$\Delta r=\;\left(r_{0}-\;2r_{45}+{\;r}_{90}\right)/2$$

(3) $$\Delta r_{p}={\;r}_{\max}-{\;r}_{\min}$$

The tensile test results were also utilized to evaluate a strain hardening exponent (n). It was calculated based on the power law describing the dependence of true stress (σ), true strain (ε) and strength coefficient (K) within a uniform elongation regime, which can be presented as follows [36]:

(4) $$\sigma =K\varepsilon^{n}$$

Finally, the Vickers hardness was measured on the RD-ND plane at a load of 2 kg using a Zwick/Roell ZHU/Z2.5 hardness tester.

## 3. Results and Discussion

### 3.1. Microstructure

**Figure 1:**
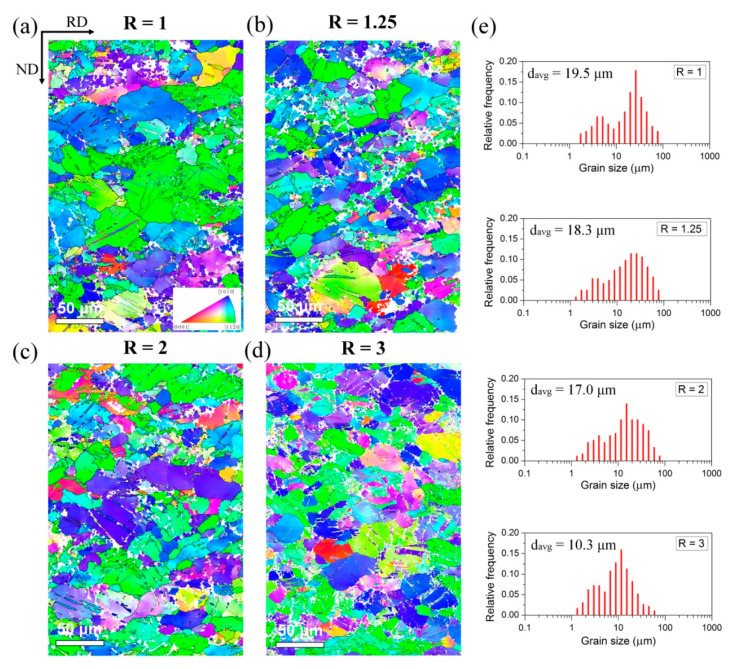
Electron backscattered diffraction (EBSD) inverse pole figure (IPF) maps of the as-rolled Mg-6Sn sheets processed at speed ratio of (**a**) R = 1, (**b**) R = 1.25, (**c**) R = 2 and (**d**) R = 3 with (**e**) grain size distributions.

**Figure 2:**
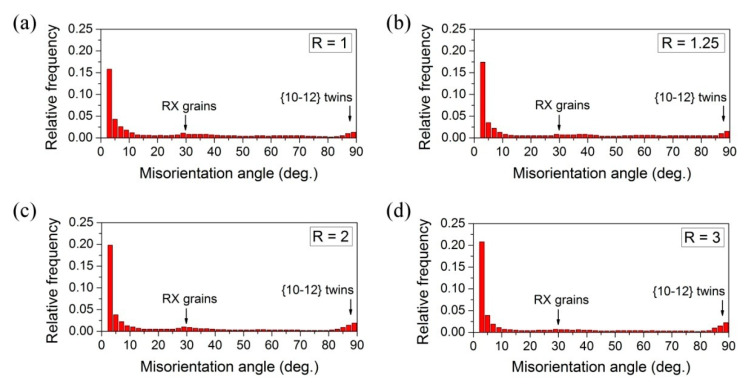
The misorientation angle distributions of the as-rolled Mg-6Sn sheets processed at speed ratio of: (**a**) R = 1, (**b**) R = 1.25, (**c**) R = 2 and (**d**) R = 3.

Figure 1 presents the microstructure of as-rolled Mg-6Sn sheets illustrated by EBSD inverse pole figure (IPF) maps observed at the RD-ND plane, while the misorientation angle distributions are shown in Figure 2. The microstructure of the conventionally rolled sheet (R = 1) is composed of a relatively coarse grains (with a size of 20–70 μm) together with a smaller fraction of newlyformed recrystallized grains of few microns as illustrated by the bimodal grain size distribution in Figure 1e. The recrystallized grains are located mainly at twin and grain boundaries (GBs) being a result of dynamic recrystallization (DRX) during hot rolling as well as static recrystallization (SRX) during reheating between passes. A slightly higher fraction of GBs with misorientation angles of around 30° (Figure 2a) corresponds to those RX grains [37,38]. A number of twins were also observed mostly in the coarse grains suggesting the activity of mechanical twinning in accommodating deformation. The weak peak in the misorientation angle distribution at about 86° indicates the activity of {10–12}<10–11> extension twinning [39,40].

It has been shown that the accumulated strain increases with increasing the speed ratio during DSR processing [41]. In turn, the higher accumulated strain induces the higher grain refinement of Mg-6Sn sheets produced by DSR in comparison to the symmetrically rolled one (Figure 1b–e). With increasing the speed ratio, the average grain size decreased—it was lowered from 19.5 μm for R = 1 to 18.3, 17.0 and 10.3 μm for R = 1.25, 2 and 3, respectively. The bimodal grain size distribution with a smaller fraction of fine RX grains is still observed for DSR-processed sheets, but the size of bigger grains has been significantly decreased. The misorientation angle distribution for DSR-processed sheets (Figure 2b–d) exhibited slightly enlarged fractions of LAGBs (especially with the misorientation angle less than 5°) and {10–12}<10–11> twins, which both increased with increasing the speed ratio.

**Figure 3:**
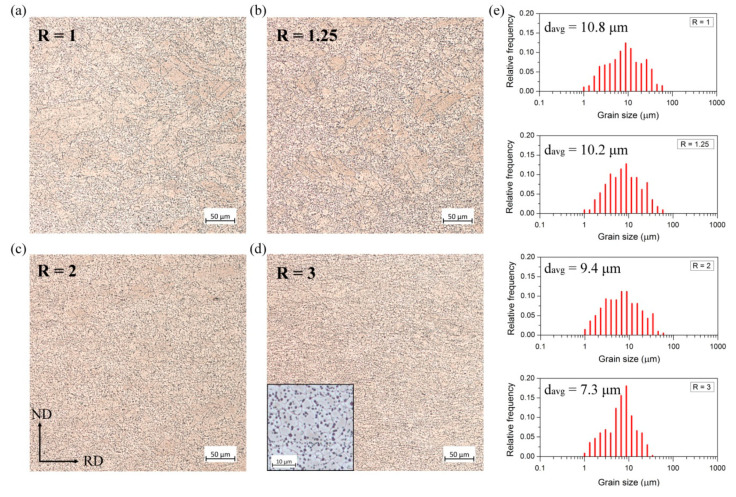
Light microscope (LM) images of the microstructure of the annealed Mg-6Sn sheets processed at speed ratio of (**a**) R = 1, (**b**) R = 1.25, (**c**) R = 2 and (**d**) R = 3 with (**e**) grain size distributions.

After annealing at 300 °C, twins disappeared and recrystallized homogeneous microstructures with equiaxed grains were generally obtained as presented in Figure 3. However, it should be noted that some elongated grains remained in the sheets processed with a low speed ratio (R = 1 and 1.25). They seem to be a remnant of coarse grains with twins observed after DSR processing (Figure 1a–b). In the case of Mg-6Sn sheets produced at R = 2 and 3, the recrystallized grains nucleated homogeneously at GBs, twin boundaries or other deformed grains with gradient strain leading to a uniform microstructure with a reduced grain size of 9.4 and 7.3 μm, respectively.

**Figure 4:**
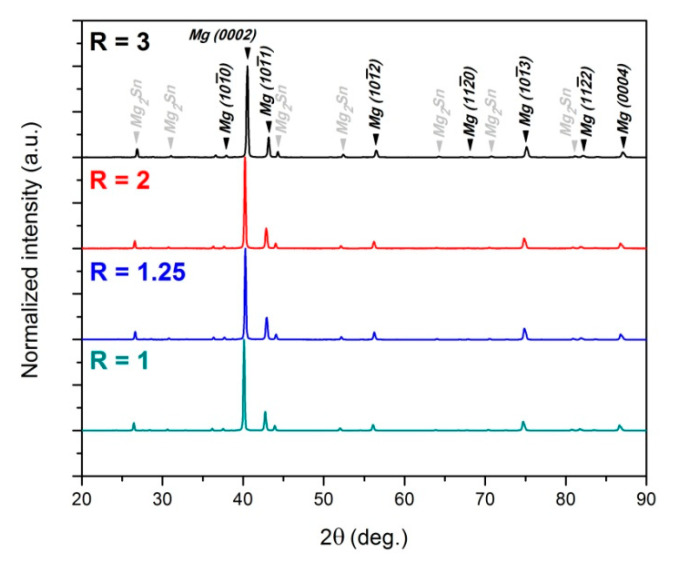
XRD patterns of as-rolled Mg-6Sn sheets processed at speed ratio of R = 1, 1.25, 2 and 3.

The metallographic observations revealed also the presence of densely distributed second-phase particles. Mg-Sn-based alloys are known to be a heat-treatable group of Mg alloys [16,42,43]. As a result of ageing treatment at 150–200 °C, Mg_2_Sn phase precipitates are formed in the shape of laths or short rods lying on the basal plane [42,43]. After annealing or plastic deformation at a higher temperature, the spherical or polygonal-shaped precipitates are rather formed [16,17], which can be observed in our work as well (see the inset in Figure 3d). Mg_2_Sn particles were also present in the as-rolled sheets. Figure 4 shows the XRD patterns for the as-rolled Mg-6Sn alloy. It is clear that, despite the strong peaks coming from Mg hexagonal lattice (especially basal plane (0002)), the additional peaks appeared at 2θ values of around 26.5, 30.7, 43.9, 52.0°, etc., corresponding to Mg_2_Sn phase [44,45]. The normalized intensities of those peaks and, thus, the volume fraction of Mg_2_Sn particles seem to be not affected by the applied speed ratio.

### 3.2. Texture

**Figure 5:**
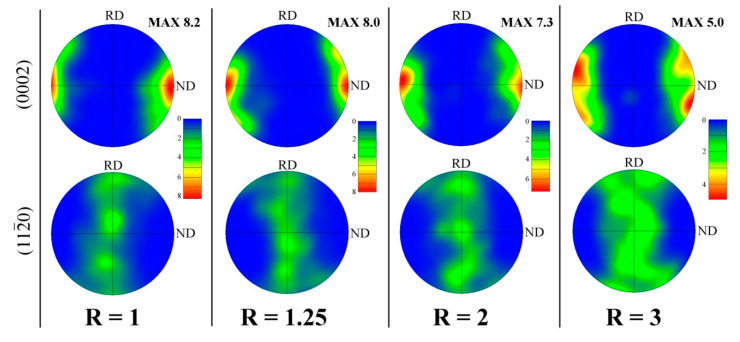
The (0002) and (10–10) pole figures of the as-rolled Mg-6Sn sheets processed with different speed ratio (R) obtained from EBSD measurements on the RD-ND plane.

The as-rolled Mg-based sheets normally exhibit a strong basal texture [2,4]. A predominant basal texture (0002)//ND also developed in the Mg-6Sn sheets. Since the EBSD measurements of the as-rolled sheets were performed on the RD-ND plane, the two dominant orientations of grains visible on the IPF maps in Figure 1 are (10–10) and (11–20) represented by blue and green color, respectively. The (0002) and (11–20) pole figures corresponding to this RD-ND plane are shown in Figure 5. A typical (0002)//ND was observed for all as-rolled sheets. However, the spread of the main fiber increased with the increasing of the speed ratio. The intensity of basal texture gradually decreased from 8.2, multiples of random distribution (MRD) for R = 1 to 8.0, 7.3 and 5.0 MRD for R = 1.25, 2 and 3, respectively. The spread of (0002) and (11–20) poles became more significant with higher speed ratio. This corresponded to the higher fraction of grains with a tilted c-axis from the ND observed in the IPF maps (represented by the purple and yellow colors). For the DSR sheet processed at R = 3, a significant splitting of basal poles towards RD can even be distinguished.

**Figure 6:**
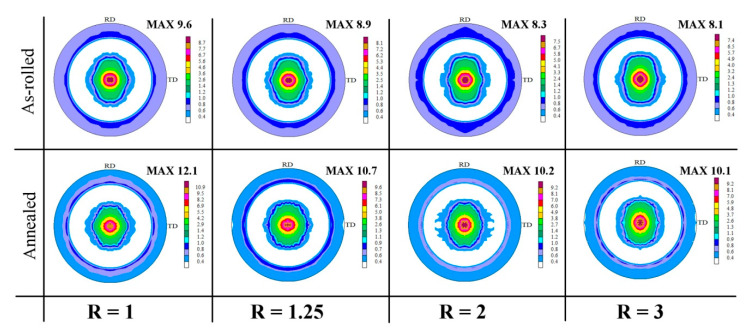
The (0002) pole figures of the as-rolled and annealed Mg-6Sn sheets processed with different speed ratio (R) obtained from XRD measurements on the RD-TD plane.

Figure 6 presents the recalculated (0002) pole figures of the as-rolled Mg-6Sn sheets collected from the RD-TD plane. All analyzed samples exhibited the basal texture, while the increasing speed ratio resulted in the gradual decrease in its intensity, i.e., the basal texture intensity was lowered from 9.6 MRD for R = 1 to 8.9, 8.3 and 8.1 MRD for R = 1.25, 2 and 3, respectively. It should be also noted that a slight splitting of basal poles has been noticed for all as-rolled samples. The (0002) basal poles were split into the TD for R = 1, 1.25 and 2, whereas for the highest R = 3 value, they seem to lean towards the RD. Generally, the hot rolling of the most common Mg-Al-Zn alloys leads to the formation of a strong single-peak basal texture [1,2,4]. The phenomenon of basal texture spreading has been discovered mainly for Mg-RE [46,47], Mg-Zn [48] or Mg-Li [47] alloys, which exhibit high ductility and formability during further deformation. The RD splitting is observed for Mg-RE as well as non-RE Mg alloys, while the TD splitting is considered unique for Mg-RE alloys [49]. Besides, the RD spreading in the basal texture has been assigned to the activation of pyramidal <c+a> slip, while the prismatic <a> slip is considered as a possible origin of the TD spreading [47,50]. Since Zeng et al. [11] proved that Sn addition leads to easier activation of the pyramidal <c+a> slip, and Wang et al. [9] showed also that the prismatic <a> slip is prone to be activated in Mg-Sn alloys, the observed RD and TD splitting is highly possible to occur due to the aforementioned reasons.

**Figure 7:**
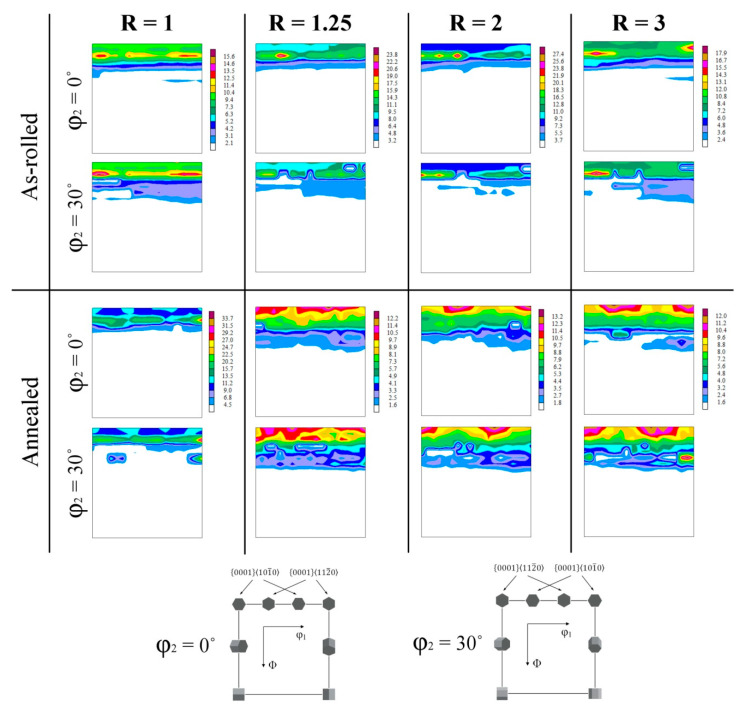
The orientation distribution functions (ODFs) (ϕ_2_ = 0° and 30° sections) of the as-rolled and annealed Mg-6Sn sheets processed with different speed ratio (R) obtained from XRD measurements on the RD-TD plane.

The main texture components present in the investigated Mg-6Sn sheets have been visualized in Figure 7 in the form of ϕ_2_ = 0° and 30° ODF sections. For easier interpretation of the results, the ideal positions for a typical basal texture component were also schematically illustrated. According to this scheme, each basal texture generates four components at ϕ_2_ = 0° and 30° sections, e.g., (0001)<10–10> texture exhibits intensity peaks at (ϕ_1_ = 0°, Φ = 0°, ϕ_2_ = 0°), (60°, 0°, 0°), (30°, 0°, 30°) and (90°, 0°, 30°). Moreover, it should be pointed out that if (0°, Φ, 0°) and (90°, Φ, 0°) texture components move to the higher value of the Φ angle, the basal planes are tilting towards the TD and RD, respectively [51].

The conventionally rolled Mg-6Sn sheet (R = 1) exhibited a relatively strong fiber extended along the ϕ_1_ = 0–90° tilted to the Φ angle of around 10°, which indicates that the basal poles were tilted by 10° from the ND. The main texture components were shifted in the ϕ_1_ angle (of ~ 5–10°) from the ideal position of both (0001)<10–10> as well as (0001)<11–20> texture components. It proves that the texture of the symmetric rolled Mg-6Sn sheet is a combination of two crystallographic orientations (0001)<10–10> and (0001)<11–20>, while the basal poles are tilted to TD and RD. Although, the strongest texture components suggest that the TD spreading dominates in the texture, which is consistent with the (0002) basal pole figure presented in Figure 6.

For the Mg-6Sn sheets processed at R = 1.25 and 2, the (0001)<11–20> texture started to dominate with the main components displayed at (30°, 10°, 0°) and (0°, 10°, 30°). The basal poles were still tilted towards TD by around 10°. In the case of DSR sheets produced at R = 3, a new strong texture component appeared at (90°, 5°, 0°), which is responsible for a slight basal pole splitting to the RD by around 5° (as presented earlier in Figure 6).

After annealing at 300 °C, the Mg-6Sn sheets also showed the basal texture; although, its intensity increased significantly in comparison to their as-rolled counterparts (Figure 6). With increasing the speed ratio, the lower intensities were noticed (i.e., 12.1, 10.7, 10.2 and 10.1 MRD for R = 1, 1.25, 2 and 3, respectively). The spread of basal texture to TD or RD observed for the as-rolled samples has been blurred. As shown in the ODF sections in Figure 7, the conventionally rolled sheet (R = 1) exhibited only the basal pole tilting. The strongest texture components at (90°, 10°, 0°) and (90°, 10°, 30°) indicated on the RD splitting of basal poles for R = 1. In the case of the DSR-processed sheets, the major fraction of (0002) poles was aligned with the ND. The texture of the annealed Mg-6Sn sheets produced at R = 1 and 1.25 combined two crystallographic orientations: (0001)<10–10> and (0001)<11–20>, whereas (0001)<11–20> texture became the dominant one for R = 2 and 3. It was manifested by four strong texture components at (30°, 0°, 0°), (90°, 0°, 0°), (0°, 0°, 30°) and (60°, 0°, 30°) which are typical for (0001)<11–20> texture.

### 3.3. Mechanical Properties

**Figure 8:**
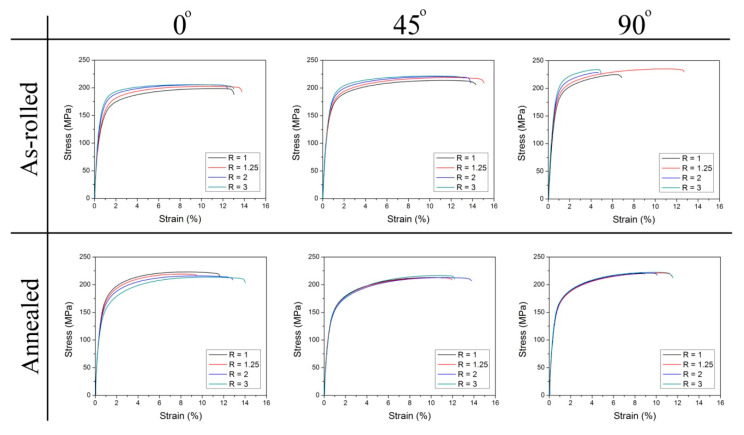
Stress–strain curves obtained for the as-rolled and annealed Mg-6Sn samples processed with different speed ratio (R).

Figure 8 shows the stress–strain curves obtained for Mg-6Sn samples cut at different angles to the RD (i.e., 0, 45 and 90°) from the as-rolled and annealed sheets, whereas the 0.2% offset yield strength (YS), ultimate tensile strength (UTS), uniform elongation (A_u_), elongation to failure (A), strain hardening exponent (n) and Vickers hardness values are summarized in Table 1. For the comparison of the overall performance of sheets processed with different speed ratio, the average value $\bar{X}$ of each parameter was calculated following the formula [52]:

(5) $$\bar{X}=\;\left(X_{0}+\;2X_{45}+{\;X}_{90}\right)/4$$

where X denotes to YS, UTS, A_u_, A or n value, and the subscript indicates an angle to the RD.

The results for the as-rolled Mg-6Sn sheets showed that the YS and UTS gradually increased with increasing speed ratio. The average value of YS and UTS for the conventionally rolled sheet (R = 1) was 141 and 215 MPa, respectively, whereas the highest strength was observed for R = 3, i.e., the mean YS and UTS of 157 and 219 MPa, respectively. The same trend was noticed for the Vickers hardness, which gradually raised from 52 HV2 for R = 1 to 55 HV2 for R = 3. The elongation to failure did not show such clear tendency. After DSR at R = 1.25, the average elongation of 13.2% was noticed, which was higher than for R = 1 (11.8%). The higher applied speed ratio resulted in the lowering of ductility to 11.9 and 10.1% for R = 2 and 3, respectively. The calculated strain hardening exponent followed the same trend. Since the higher n values are beneficial to enhance the ability to uniform plastic deformation, the higher uniform elongation correlated strictly with increasing strain hardening exponent. It should be also pointed out that the calculated n values of around 0.1–0.13 are close to those of the popular AZ31 alloy processed by DSR (*n* = 0.09–0.16 [53]). They are not as high as for Mg-Re alloys (*n* = 0.24–0.29 [46]) or a dilute Mg-0.4Sn-0.7Y alloy sheets after hot rolling and annealing (*n* = 0.27–0.35 [12]). Although, it should be stressed that the strain hardening exponent decreases gradually with increasing Sn content. Zhao et al. [54] reported for as-extruded Mg-Sn alloys with 1, 2, 3 and 4 wt.% of Sn that n value can be reduced from 0.21 for Mg-1Sn to 0.13 for Mg-4Sn. Finally, all analyzed sheets exhibited a significant anisotropy of strength and plasticity (i.e., higher YS and UTS with reduced elongation were observed at 90° direction as compared to 0°).

The microstructure and texture characteristic based on the EBSD measurements has been presented in Table 2 to discuss the obtained tensile test results. It summarizes the average grain size, the fraction of LAGBs with a misorientation angle less than 5° and {10–12} extension twins, maximum basal texture intensity and calculated Schmid factor (SF) for the basal <a> slip for loading along RD and TD. It is clear that the increased YS and UTS result mainly from the decreased grain size (according to the Hall–Petch relationship [55]) as well as the increased amount of LAGBs, corresponding to the low angle dislocation boundaries and, thus, the higher dislocation density [29]. It should be mentioned that the room temperature deformation of Mg alloys is mainly attributed to the activity of basal <a> slip and {10–12}<10–11> twinning, while other slip systems exhibits a few times higher CRSS [4,5]. Based on the Schmid law [4], the higher SF for basal slip lead to easier deformation, but the results obtained for the as-rolled sheets do not follow this rule completely (Table 1 and Table 2). The higher spread of basal texture and lower maximum intensities noticed for higher speed ratios (Figure 5) caused that the microstructure became more preferentially oriented for the basal slip at higher speed asymmetry. The SFs along RD and TD both increased at higher R values, but they did not contribute to increasing the ductility. The elongation to failure raised for the low asymmetry ratio (R = 1.25), while further increasing of the speed ratio caused the diminishment of the plasticity. It suggests that the deformation ability of sheets processed at R = 2 and 3 was reduced by the higher dislocation density and fraction of {10–12} twins. It is known that twin boundaries may act as barrier to a slip movement and become the crack sources [5,56]. After twinning deformation, a significant residual stresses remain in {10–12} twins [57], which can be estimated based on nanoindentation [58]. This residual stresses may further lead to easier activation of detwinning and pronounced crack nucleation [57]. Finally, the calculated SF values explained the higher YS but lower elongation along TD (90°) in respect to the RD (0°). The SF along TD was lower for all analyzed sheets as compared to the RD.

Table 1 summarizes also the mechanical properties of annealed Mg-6Sn sheets. It is clear that the applied heat treatment caused a decrease in YS and hardness due to recovery and recrystallization processes. The UTS remained at the same level as for the as-rolled sheets. Surprisingly, the elongation to failure was slightly decreased. The only exception is the DSR sheets processed at R = 3, which exhibited a rise in average elongation from 10.1 to 12.1%. The plasticity reduction for R = 1, 1.25 and 2 may result from the increased intensity of the basal texture after annealing (Figure 6). Besides, the microstructure of the analyzed sheets comprised a lot of Mg_2_Sn precipitates located at grain boundaries, which may restrict the enhancement of plasticity [16]. Nevertheless, it should be pointed out that the uniform elongation, elongation to failure and strain hardening exponent increased with increasing the speed ratio, which resulted from the gradually lowered intensity of the basal texture (as shown in Figure 6) and more homogeneous microstructure after annealing observed for the DSR-processed sheets (Figure 3). The n values for all annealed Mg-6Sn sheets raised significantly from 0.1–0.13 (observed for as-rolled sheets) up to 0.15–0.16 and becamecloser to values obtained for the hot rolled and annealed Mg-0.4Sn-0.7Y alloy reported by Wang et al. [12]. The more homogeneous and refined microstructure after annealing should also give a rise to higher fracture toughness of the obtained Mg-6Sn sheets. As shown by Somekawa and Mukai [59], the grain size refinement leads to higher fracture toughness of Mg and a limited activity of twins, which normally become a path for crack propagation. Besides, the weakened and randomized basal texture of the DSR-processed sheets may additionally improve their fracture toughness. It has been shown that the fracture toughness is highly dependent on the loading direction with respect to the crystallographic orientation of grains [60,61]. The fracture toughness of the AZ31 alloy is higher when a crack propagates perpendicularly to the basal planes as compared to the parallel direction [60]. Thus, the weakening and spreading of basal texture should improve fracture toughness of the DSR-processed sheets for cracks propagating in the rolling plane.

What is more important is that the annealing at 300 °C affected the anisotropy of mechanical properties of the Mg-6Sn sheets. Table 3 presents the normal anisotropy r_m_, planar anisotropy Δr and earing tendency Δr_p_ estimated for the as-rolled and annealed sheets. It should be stressed that the r_α_ value for an isotropic material is 1, r_α_> 1 means that the material is resistant to thinning, while r_α_< 1 implies that the material will be reduced more in the thickness than in the width [62]. In general, the rolled AZ31 sheets with a strong basal texture exhibit the Lankford parameter r-values as high as 3 due to a small thickness–direction strain [12,18]. When the basal planes are aligned with the tensile direction, the basal <a> and prismatic <a> slips cannot accommodate the thickness–direction strain. The only deformation mechanisms that can accommodate strain in the c-axis of Mg lattice are pyramidal <c+a> slip or twinning systems. Due to the high CRSS of pyramidal slip, it is hardly activated at room temperature [2,4,5]. Table 3 shows that the as-rolled and annealed Mg-6Sn sheets were characterized by a very low Lankford parameter. In the case of the as-rolled sheets, the normal anisotropy r_m_ was in the range from 1.14 to 1.39. It decreased after annealing to values close to 1, which indicated a very uniform deformation of tensile samples during testing. Such low normal anisotropy has been observed so far for Mg-RE alloys showing extremely high ductility and formability [46,49]. Wang et al. [12] reported also the low r-value of 1.52 for hot rolled and annealed Mg-0.4Sn-0.7Y sheets. Considering the texture results obtained in the current work (Figure 5, Figure 6 and Figure 7), the low normal anisotropy can be attributed to the splitting of basal texture to TD and RD and, thus, easier activation of basal slip and twinning to accommodate the c-axis strains. Another important parameter is the planar anisotropy Δr and earing tendency Δr_p_, which shows the spread of Lankford parameters for different loading directions. The most expected Δr value for the deep drawing is ~0, when Δr > 0 earing forms at RD/TD positions, while for Δr < 0, they are shifted to 45° [62]. The calculated Δr_p_ values for the as-rolled Mg-6Sn sheets exceeded 1, whereas the significantly lower values in the range of 0.28–0.59 were observed for annealed sheets. The planar anisotropy Δr was lowered as well. After rolling, it was in the range of 0.22–0.51, while Δr values close to 0 (ranging from −0.04 to 0.20) were observed after annealing. All above mentioned results indicated that the obtained Mg-6Sn sheets were characterized by a very low anisotropy, which is generally not achieved in the case of the most common Mg-Al-Zn alloys.

## 4. Conclusions

In the present work, the effect of the shear deformation introduced by DSR processing on the microstructure, texture and mechanical properties of Mg-6Sn alloy was investigated. The level of introduced shear stresses during DSR was controlled by adjusting different speed ratio between upper and lower rolls ranging from R = 1 to 3. The main conclusions can be summarized as follows:

1. DSR processing resulted in the pronounced grain refinement of Mg-6Sn sheets and spreading of basal texture as compared to the conventionally rolled one. The average grain size and basal texture intensity gradually decreased with increasing the speed ratio.
2. The texture of the symmetrically rolled Mg-6Sn sheet was a combination of two crystallographic components (0001)<10–10> and (0001)<11–20>, while the (0001)<11–20> texture component became the dominant one for DSR processed sheets (especially for higher speed ratio). Moreover, the basal poles splitting to TD or RD have been observed for all analyzed Mg-6Sn sheets (they were tilted to the TD for R = 1, 1.25 and 2, whereas for the highest R = 3 value they leant towards the RD).
3. For the as-rolled Mg-6Sn sheets, YS and UTS increased gradually with increasing speed asymmetry ratio. A significant anisotropy of strength and ductility between RD and TD has been observed as well. After annealing at 300 °C, the Mg-6Sn sheets became more homogeneous. The elongation to failure was increased with higher speed ratios resulting from the weakened basal texture intensity.
4. The as-rolled and annealed Mg-6Sn sheets were characterized by a very low normal and planar anisotropy values, which are generally not achieved in the case of the most common Mg-Al-Zn alloys. The normal anisotropy of the annealed sheets was in the range of 0.91–1.16.

## Figures and Tables

**Table 1:** Mechanical properties (YS—yield strength, UTS—ultimate tensile strength, A_u_—uniform elongation, A—elongation to failure, *n*—strain hardening exponent) of the as-rolled and annealed Mg-6Sn sheets processed with different speed ratio.

State	Speed Ratio	Direction	YS (MPa)	UTS (MPa)	A_u_ (%)	A (%)	*n*	$\bar{\mathrm{YS}}\;(\mathrm{MPa})$	$\bar{\mathrm{UTS}}\;(\mathrm{MPa})$	$\bar{A_{u}}\;(\%)$	$\bar{A}$	$\bar{n}$	Hardness (HV2)
As-rolled	R = 1	0°	128 ± 14	200 ± 3	10.3 ± 0.2	13.6 ± 1.6	0.12 ± 0.01	141	215	9.5	11.8	0.12	52 ± 1
		45°	138 ± 5	216 ± 4	10.8 ± 0.4	13.7 ± 0.5	0.12 ± 0.01						
		90°	160 ± 2	226 ± 1	5.9 ± 0.6	6.2 ± 0.4	0.12 ± 0.01						
	R = 1.25	0°	128 ± 2	202 ± 5	10.7 ± 0.9	14.8 ± 1.5	0.12 ± 0.01	143	217	10.7	13.2	0.13	53 ± 1
		45°	143 ± 4	216 ± 3	11.2 ± 0.5	13.9 ± 0.7	0.13 ± 0.01						
		90°	156 ± 1	233 ± 2	9.5 ± 1.1	10.3 ± 1.7	0.13 ± 0.01						
	R = 2	0°	132 ± 11	201 ± 5	10.4 ± 0.8	13.8 ± 2.0	0.11 ± 0.01	146	217	9.5	11.9	0.11	54 ± 1
		45°	144 ± 4	217 ± 3	11.0 ± 0.9	14.0 ± 1.1	0.12 ± 0.01						
		90°	165 ± 6	231 ± 7	5.4 ± 2.5	5.9 ± 3.2	0.11 ± 0.01						
	R = 3	0°	143 ± 6	203 ± 3	8.6 ± 0.5	12.1 ± 0.3	0.10 ± 0.01	157	219	7.9	10.1	0.10	55 ± 1
		45°	155 ± 2	218 ± 3	9.0 ± 1.5	11.4 ± 2.9	0.10 ± 0.01						
		90°	173 ± 2	235 ± 2	4.9 ± 2.7	5.4 ± 3.1	0.10 ± 0.01						
Annealed	R = 1	0°	145 ± 3	220 ± 3	7.9 ± 0.5	11.4 ± 1.5	0.13 ± 0.01	135	216	9.0	10.9	0.15	47 ± 2
		45°	128 ± 3	213 ± 1	10.3 ± 0.5	11.9 ± 1.4	0.16 ± 0.01						
		90°	140 ± 3	219 ± 5	7.6 ± 2.0	8.5 ± 3.0	0.15 ± 0.01						
	R = 1.25	0°	139 ± 4	216 ± 4	7.8 ± 0.4	9.6 ± 0.6	0.14 ± 0.01	134	217	9.1	11.1	0.15	47 ± 2
		45°	127 ± 4	215 ± 2	9.9 ± 0.3	12.4 ± 0.9	0.16 ± 0.01						
		90°	141 ± 1	220 ± 3	8.9 ± 1.9	9.8 ± 2.5	0.16 ± 0.01						
	R = 2	0°	132 ± 3	214 ± 2	9.3 ± 0.3	12.2 ± 0.5	0.15 ± 0.01	130	215	9.6	11.6	0.16	47 ± 1
		45°	126 ± 2	213 ± 3	10.7 ± 0.1	13.0 ± 0.7	0.16 ± 0.01						
		90°	136 ± 1	219 ± 2	7.7 ± 0.1	8.3 ± 1.1	0.15 ± 0.01						
	R = 3	0°	124 ± 2	214 ± 1	10.1 ± 0.4	14.0 ± 1.1	0.17 ± 0.01	128	218	9.7	12.1	0.16	47 ± 1
		45°	125 ± 2	218 ± 2	10.1 ± 0.2	12.5 ± 0.8	0.17 ± 0.01						
		90°	136 ± 8	222 ± 1	8.4 ± 1.0	9.5 ± 1.6	0.15 ± 0.01						

**Table 2:** Microstructure and texture characteristic of the as-rolled Mg-6Sn sheets based on EBSD.

Speed Ratio	Grain Size (μm)	3–5° LAGBs Fraction (%)	{10-12} Twins Fraction (%)	Texture Intensity	SF Along RD	SF Along TD
R = 1	19.5	15.8	2.8	8.2	0.22	0.19
R = 1.25	18.3	17.4	3.0	8.0	0.24	0.21
R = 2	17.0	19.8	4.2	7.3	0.23	0.22
R = 3	10.3	20.8	4.7	5.0	0.28	0.24

**Table 3:** Normal anisotropy r_m_, planar anisotropy Δr and earing tendency Δr_p_ of the as-rolled and annealed Mg-6Sn sheets processed with different speed ratio.

State	Speed Ratio	Direction	Lankford Parameter	r_m_	Δr	Δr_p_
As-rolled	R = 1	0°	0.79	1.17	0.35	1.11
		45°	0.99			
		90°	1.90			
	R = 1.25	0°	0.72	1.14	0.22	1.06
		45°	1.03			
		90°	1.78			
	R = 2	0°	0.83	1.35	0.51	1.56
		45°	1.10			
		90°	2.38			
	R = 3	0°	0.76	1.39	0.39	1.66
		45°	1.20			
		90°	2.42			
Annealed	R = 1	0°	0.91	1.14	0.13	0.59
		45°	1.07			
		90°	1.50			
	R = 1.25	0°	1.00	1.16	−0.04	0.28
		45°	1.19			
		90°	1.28			
	R = 2	0°	0.82	0.91	0.20	0.39
		45°	0.81			
		90°	1.20			
	R = 3	0°	0.76	1.03	−0.05	0.49
		45°	1.05			
		90°	1.25			

## Data Availability

The data presented in this study are available on request from the corresponding author.
